# Factors affecting the levels of protection transferred from mother to offspring following immune challenge

**DOI:** 10.1186/1742-9994-11-46

**Published:** 2014-07-01

**Authors:** Christina M Coakley, Vincent Staszewski, Katherine A Herborn, Emma JA Cunningham

**Affiliations:** 1 Centre for Infection, Immunity and Evolution, Institute of Evolutionary Biology, School of Biology, King’s Buildings, University of Edinburgh, Edinburgh EH9 3JT, UK

**Keywords:** Maternal antibodies, Maternal effect, Trade-offs, Immunity, Differential allocation, Ecoimmunology

## Abstract

**Introduction:**

The transfer of antibodies from mother to offspring is key to protecting young animals from disease and can have a major impact on responses to infection and offspring fitness. Such maternal effects also allow young that may be exposed to disease in early life to focus resources on growth and development at this critical period of development. Maternally transferred antibodies are therefore an important source of phenotypic variation in host phenotype as well as influencing host susceptibility and tolerance to infection across generations. It has previously been assumed the transfer of antibodies is passive and invariant and reflects the level of circulating antibody in the mother at the time of transfer. However, whether females may vary in the relative amount of protection transferred to offspring has seldom been explored.

**Results:**

Here we show that females differ widely in the relative amount of specific blood antibodies they transfer to the embryonic environment (range 9.2%-38.4% of their own circulating levels) in Chinese painted quail (*Coturnix chinensis*). Relative transfer levels were unrelated to the size of a female’s own immune response. Furthermore, individual females were consistent in their transfer level, both across different stages of their immune response and when challenged with different vaccine types. The amount of antibody transferred was related to female condition, but baseline antibody responses of mothers were not. However, we found no evidence for any trade-offs between the relative amount of antibody transferred with other measures of reproductive investment.

**Conclusions:**

These results suggest that the relative amount of antibodies transferred to offspring can vary significantly and consistently between females. Levels of transfer may therefore be a separate trait open to manipulation or selection with potential consequences for offspring health and fitness in both wild and domesticated populations.

## Introduction

The immune response plays a key role in protecting individuals from disease and this immune defense can be extended to offspring via the transfer of maternal immunity (for review see [1]). Maternal antibodies are produced when mothers are exposed to pathogens in the environment prior to, or during, reproduction but can also be induced by health interventions such as vaccination. The resulting maternal immunity can be both crucial in protecting offspring from disease during early development and in allowing offspring to focus more resources on growth and less on dealing with pathogen exposure during this critical period of development [2]. Maternally-transferred antibodies are therefore an important source of phenotypic variation in a range of offspring traits as well as influencing responses to infection across generations. Such maternal effects, whereby female condition or allocation of resources during pregnancy or egg laying can influence the phenotype of the developing young are now recognized to have a major impact on the relationship between genotype and fitness [3]: Maternal immunity may therefore have both a direct impact on offspring health and fitness and be a powerful selective pressure that can accelerate or limit how host and pathogen traits might change in response to selection [4].

While there has been much work on factors that affect an individual’s immune response, far less is known about factors affecting the level of antibody transferred from mothers to offspring between and within individuals over time. The maternal effect of transferring immunity to offspring is widespread throughout different animal groups including mammals, birds, fish and invertebrates [5]. In mammals these substances can be transferred directly from the mother prenatally through the placenta and post-natally through the colostrum (first milk) and continued milk [6]. In birds, most substances are transferred to offspring via the yolk when the egg is in oogenesis [7] prior to other egg components such as the albumen and shell being added. In both these groups, the transfer of protective antibodies can be induced by maternal vaccination and this has been harnessed as strategy to protect young from certain disease causing pathogens e.g. whooping cough in humans [8] and infectious bursal disease (IBD) in chickens [9]. Maternally transferred protection has previously been assumed to be less important in other taxa but ecoimmunology studies have demonstrated similar protection strategies, though these may stem from different types of mechanism [10].

It has previously been assumed that the transfer of maternal immunity is passive and that high antibody responders simply transfer more to their offspring by default [10]. In birds, much of the early literature from poultry science suggests the level of antibody transfer is invariant and that females consistently transfer approximately 20% of their circulating antibody level to each egg produced [11]. This would suggest that simply selecting for high responders would also select for females that transfer a high level of antibodies to their offspring. However, variation in the transfer part of the process has seldom been fully explored at the individual level and whether variation may exist between individuals in the ratio of their circulating antibodies transferred to offspring, or indeed how this relates to the production of their own immune response, remains unclear. Given independent mechanisms are responsible for the production and transfer parts of the process [10] the level of transfer may itself be a trait open to selection [5] or manipulation. Furthermore, recent literature from the field of evolutionary ecology has found that levels of immune transfer may vary in response to a number of factors including mating partner [12], sex of offspring [13] or position in a clutch [14]. This is consistent with life history theory, which would predict that the levels of antibody transferred to different offspring should vary, depending on the likelihood of their success. However, it remains unclear as to whether levels of antibody transferred are actively targeted to different offspring or are simply a consequence of changing levels of antibodies in the mother over time as different eggs in a laying sequence are formed. This latter possibility does not exclude these mechanisms from being adaptive but whether plastic adjustment may also occur, and what factors, if any, may induce such adjustment, remains unclear.

Establishing how patterns of antibody allocation relate to a female’s antibody production within and between individuals is key to establishing the implications of these maternal effects and how they might be usefully applied in the protection of young from disease. However, progress has been hampered by a lack of information on how a mother’s antibody level varies with levels of allocation to the embryonic environment over the same period as eggs or young are being formed as antibody levels are unlikely to be consistent in a mother over this time. Data from the general immunological literature would suggest that the typical pattern of an antibody response post-challenge is characterised by a slow then increasing rise to peak antibody level, followed by a steady decline as antibodies are catabolised [15]. After acute infection, cells produced by the primary immune response persist as memory cells allowing rapid production of further antibodies to be produced if there is re-infection. Co-infection is common with females potentially producing a range of specific antibodies to a range of different types of pathogen they encounter in the environment [16]. If mounting an immune response is costly, the transfer of large numbers of antibodies to offspring is therefore likely to be an expensive process for the mother, even if the process of transfer is entirely passive. The ability to transfer essential constituents to the developing offspring may therefore be expected to relate to a mothers condition and/or result in trade-offs between other life history traits associated with reproduction or survival [17].

Here we investigate experimentally in Chinese painted Quail (*Coturnix chinensis*) whether there is variation between individual females in the amount of antibodies transferred to their offspring and whether individual females are consistent in the ratio of antibodies passed to their different eggs produced over the course of an antigen challenge and between different types of challenge (a bacterial and viral challenge). We then investigate whether any female traits co-vary with their transfer ability and discuss the potential implications of this variation on evolutionary and ecological processes affecting host parasite responses and how such variation might be usefully harnessed in an applied context.

## Results

### Variation between females in immune responses and levels of transfer

We challenged females (n = 38) at the start of egg laying with one of two inactive vaccines; one viral vaccine against Newcastle Disease virus (Nobilis Paramyxo P201) (n = 19) and one bacterial vaccine against salmonella (Nobilis SalenvacT), n = 19). We then monitored maternal and egg antibody levels over the next 42 days. Of the 38 challenged females, egg sequences that had corresponding maternal sequences were collected for 21 females (n = 7 NDV and n = 14 Salmonella). These females had been reared in a pathogen free environment and had no previous exposure to disease.

**Figure 1:**
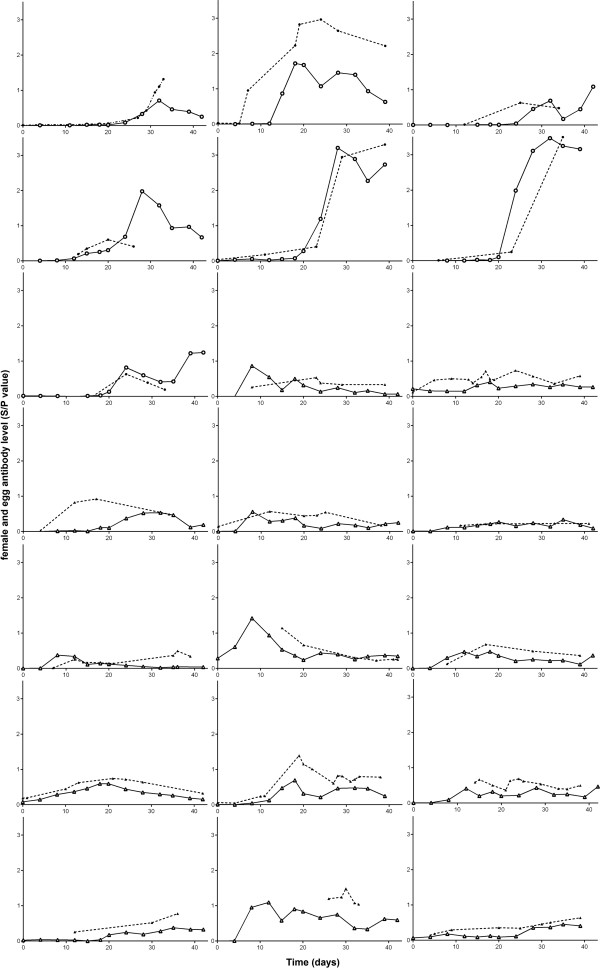
**Individual female and egg antibody levels graphed over time for n = 21 females.** Solid line and open points indicate female antibody concentration (triangles- NDV vaccinated, circles- SalenvacT vaccinated). Dotted line and solid points indicate egg antibody concentration (triangles – NDV vaccinated, circles- SalenvacT vaccinated).

Post challenge, females showed a steady increase in antibody level in the days that followed until peak antibody level was reached, followed by a slower decline in antibody level over time (Figure 1). The type of challenge did not influence the timing of peak response; F_1,20_ = 2.37, p = 0.149 but across both challenge types females varied both in the time they took to reach their peak of circulating antibody level (mean peak day 22.22 ± 3.425_SE_, n = 21) and in the magnitude of their response (mean peak size 1.127 ± 0.163_SE_).

**Figure 2:**
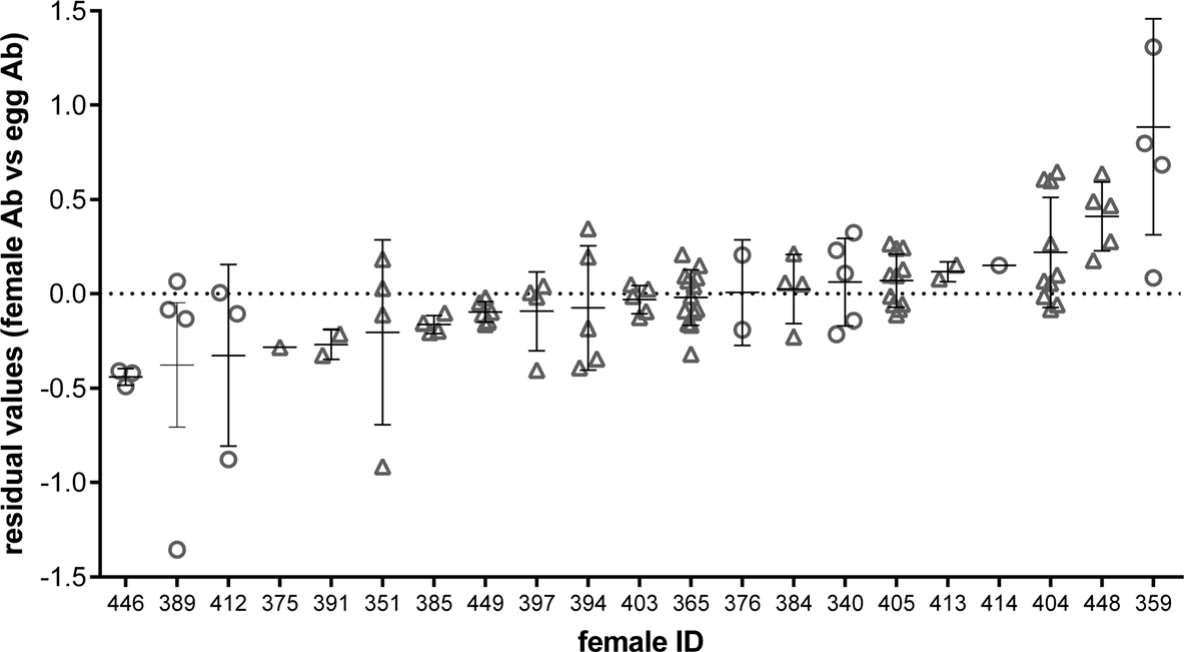
**Inter-individual variability in the capacity to transfer antibodies.** Relationship between residual of the relationship between female and egg antibodies (triangles- NDV vaccinated females, circles- SalenvacT vaccinated females). Each point indicates the residual relationship between female antibody and a single egg at a certain point in time.

Maternal patterns of antibody production were reflected in the level of antibodies found in eggs produced over the same time period (Figure 1). Both the number of days post challenge and maternal antibody level per day, therefore had a large effect on the level of antibody present in the eggs laid at the corresponding time (Table 1). However there was significant variation around this relationship suggesting other factors may influence the relative level of transfer from mother to egg over and above this response. Therefore, to examine whether females differed in the relative level of antibodies they transferred to their eggs when differences in antibody level due to time were controlled for, a comparison between the models with and without female ID as a random effect was explored. Female ID play a significant role in the model (using log likelihood ratio); χ^2^_1_ = 25.18, p = <.0001. Within females, the repeatability of the amount transferred over the course of the laying sequence was high, both when we controlled for day of sampling (intraclass correlation coefficient); r = 0.728 (Figure 2) and when day of sampling was not included in the model; r = 0.688. Assuming a female’s blood volume equals 10% of her body weight [18] the results suggest that females transfer on average 25.5% of their circulating antibody level to each egg which is, in line with previous findings in poultry [10]. However, the variation between females was high with the lowest responders transferring an estimated 9.21% (range 7-10%) and the highest transferring 38.4% (range 22-58%).

**Table 1:** Summary of linear mixed-effects model of egg antibody titre (log)

**Response**	**Explanatory**	***df***	**F**	***p***
Yolk Ab (log)	**Female Ab (log)**	1	47.85	**<.0001**
	Female mean condition	1	0.94	0.345
	Yolk mass	1	2.42	0.124
	Egg investment	1	0.88	0.360
	Treatment	1	2.65	0.121
	Female peak Ab	1	0.05	0.814
	Time (day)	1	2.07	0.154
	**Time (day)**^**2**^	1	6.65	**0.012**
	**Interaction**			
	Female Ab (log)*Female mean condition	1	11.09	**0.004**

Results for a linear mixed effects model on egg antibody titre. Significant fixed effects are in bold.

While there was a strong relationship between egg antibody levels and female antibody levels on specific days (Table 1), the mothers with the highest overall response did not transfer more to their eggs overall and peak antibody level of mother had no relationship with egg antibody levels through time (Table 1). Higher antibody responders therefore do not necessarily transfer more antibodies to their offspring due to the high levels of variation between females in their relative transfer rates.

### Effects of maternal traits and maternal investment on maternal Ab level, egg Ab level and relative transfer level

**Figure 3:**
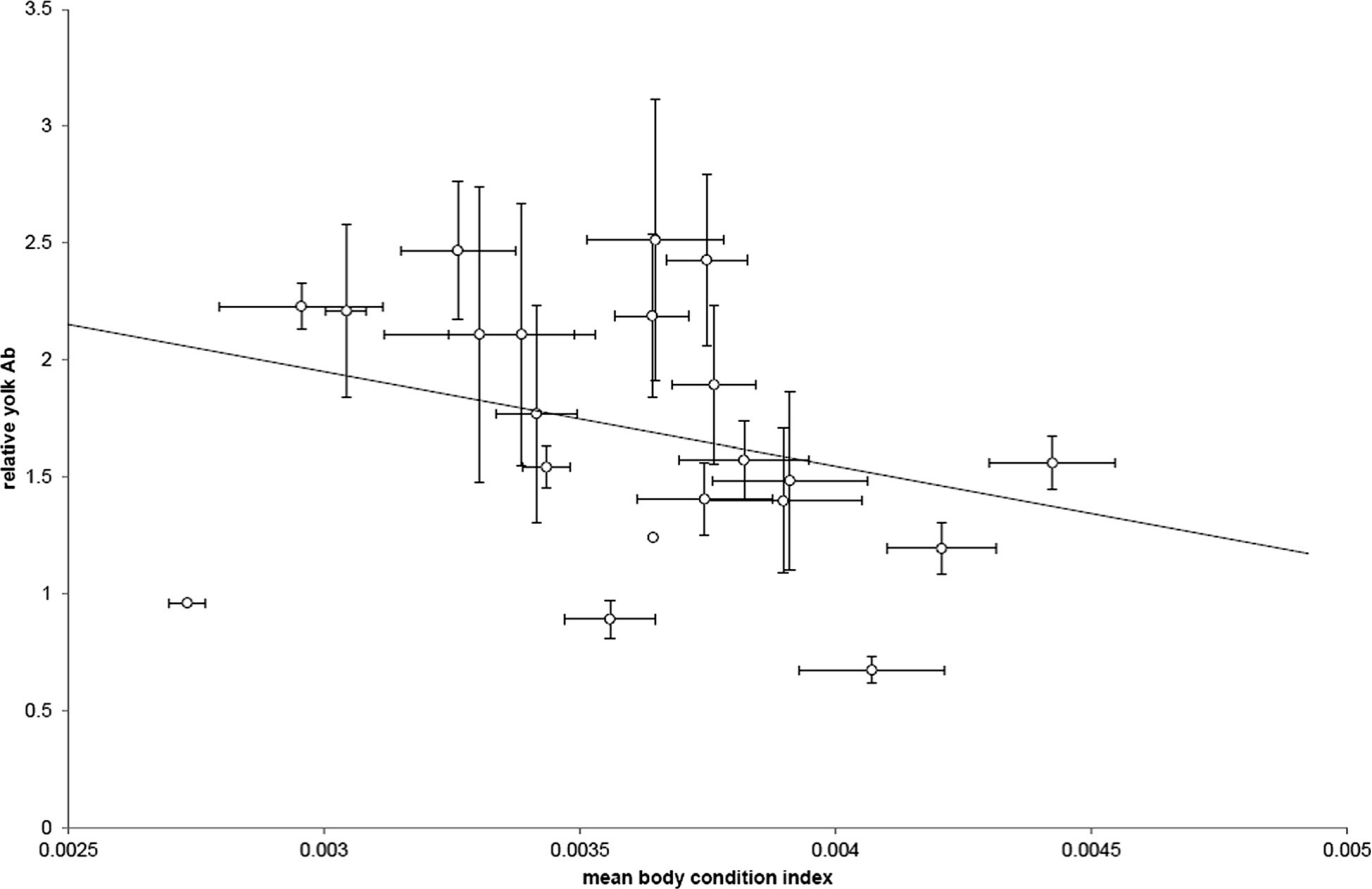
**Egg antibody level and female body condition.** Relative transfer of female antibodies transferred to eggs in relation to female’s mean body condition.

We then investigated the relationship between female immune responses and the relative amount of antibodies transferred with other female traits. We had selected non-live vaccines for the experiments to ensure any effects we detected were only related to the effect of producing the associated immune response, without any costs associated with the pathology caused by parasitism or infection which, by definition, incurs a direct cost to the host. We found no direct effect of between maternal condition on maternal circulating antibody levels or egg antibody level (Table 1) (or any of the other measures of maternal antibody response (all p > 0.1). However, we found a significant interaction between maternal antibody level and maternal condition on the level of antibodies transferred to a female’s eggs (Table 1). This was found to be because females in lower body condition transferred a greater proportion of their circulating antibodies to their eggs than females in better condition (Figure 3). No significant relationship was found between the amount of antibody transferred and total egg investment over the period of the experiment, suggesting that females who were transferring a greater ratio of their antibody level were not trading off costs associated with a higher transfer with resources allocated to reproduction in the same time frame. Mean eggs investment also had no relationship on the propensity to transfer antibodies to the egg (Table 1). These findings are confirmed by examining a model testing the effects of these different factors on the proportion of antibody transferred directly (Table 2).

**Table 2:** Linear mixed-effects model of ratio of antibodies transferred to eggs

**Response**	**Explanatory**	***df***	**F**	***p***
Relative	Yolk mass (g)	1	0.46	0.641
	**Female mean condition**	1	−2.45	**0.024**
	Female peak Ab	1	0.37	0.705
yolk Ab				
	Treatment	1	1.21	0.243
	Time (day)	1	0.197	0.844

Results for a linear mixed effects model on relative transfer of antibodies. Significant fixed effects are in bold.

### Maternal, egg and relative transfer levels in response to different and subsequent challenge types

**Figure 4:**
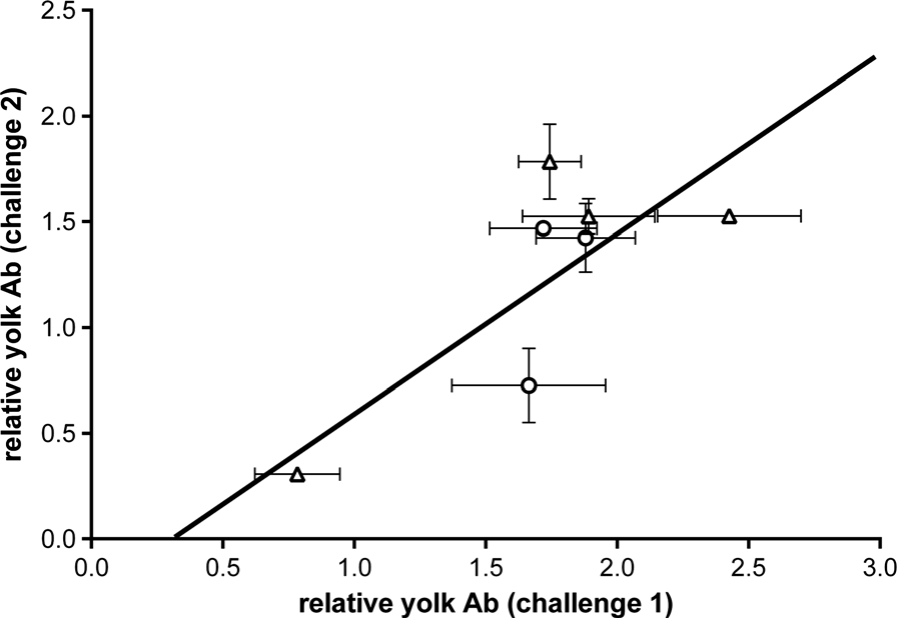
**Positive correlation between mean ratio of antibodies transferred to eggs from females (n = 7) challenged twice.** Circles represent females vaccinated with SalenvacT first, triangles represent females vaccinated with NDV first.

A subset of females was challenged again at day 42 to look at the repeatability of transfer patterns across different challenge types: individuals that had been in the bacterial challenge group (SalenvacT group) were challenged with the viral NDV vaccine (n = 4) and individuals from the viral challenge group (NDV group) were challenged with the bacterial vaccine (SalenvacT) (n = 3). In these individuals we found a positive relationship between the relative level of antibodies transferred to eggs in response to the first challenge and the relative level transferred in response to the second challenge, irrespective of which treatment was given first: F_1,6_ = 7.287, p = 0.042 (Figure 4). Again, using the log likelihood ratios (between models with and without female ID as a random effect), there was significantly more variation between individuals (χ^2^_1_ = 6.07, p = 0.01) than within individuals and the estimate of across challenge repeatability (r = 0.577) was again high.

## Discussion

Here we show that while females vary in the timing and strength of their antibody production following a standard antigen challenge, the relative amount of their own antibodies that individual females transfer to eggs over the course of this variable immune response is highly consistent. However, some females consistently transfer more than others. Furthermore, the proportion of antibody transferred by a particular female appeared to be consistent across two different challenge types which utilize different arms of the immune response (a bacterial and viral challenge). The ratio of circulating antibody that was transferred to the egg was independent of the individual’s overall strength of the antibody response highlighting that transfer mechanisms can vary independently of production levels. The amount of antibody transferred was negatively related to the female’s body condition, but the female’s circulating antibody level prior to transfer was not. However, we found no evidence for any trade-off between levels of allocation of immunity to the egg or the female’s circulating antibody level with overall levels of reproduction.

The results from this study demonstrate that a female’s capacity to transfer antibodies to her eggs is not just related to her ability to produce an antibody response. This has important implications as it suggests that both a female’s propensity to produce a particular level of immune response and her ability to transfer it to offspring may be two separate traits open to manipulation or selective processes. In natural populations, the fitness benefits of how protection is allocated between a mother investing in her own immune response and that of her offspring is likely to depend on a number of factors, including the condition a mother finds herself breeding in, the likely pathogen exposure faced and the likelihood a female may breed again in the future as they balance the costs and benefits of allocation strategies over their life history. However, ultimately, any variation in the level of transfer will create a more variable environment for pathogens and therefore impact any ensuing arms race between host and parasite.

A separation of the mechanisms responsible for the generation and transfer of an immune response is also potentially important commercially. Poultry lines, for example, have been established with genetic differences in adult antibody responses to select for genotypes that produce higher levels of antibody [19,20]. If the level of antibody transfer to offspring could separately be selected upon for either high or low transfer levels, this could potentially be advantageous; high transfer levels may be advantageous for some purposes, for example, in the production of antibodies in chicken eggs for therapeutic purposes or in flocks where maternal vaccination to transfer protection to offspring is important in offspring health (whereby mothers are vaccinated to confer protection to offspring against common diseases as a cost effective alternative to vaccinating all offspring in many poultry systems). However, low transfer levels may be advantageous in other situations e.g. *in ovo* vaccination strategies are a common strategy for protection against some diseases and successful uptake by the developing embryo requires maternal antibodies to be low. For example, it is well documented that maternal antibodies impede vaccination procedures for Marek’s disease, and can reduce its protection by up to 39% [21].

Our study suggested females are highly repeatable in their transfer levels and that there may be consistency in antibody transfer across two vaccine types suggesting generality of the trait and that it is not pathogen specific. Between individual variation estimates and within-individual repeatability estimates for traits can be extremely useful in estimating the potential heritability of traits and their potential to evolve [22]. While some studies have shown genetic differences between domestic lines of chicken in Ab production [23] and there is some evidence that genetically different color morphs of pigeon may transfer different amounts of maternal Ab [24], to our knowledge there is very little information on relative between- and within-individual variability in levels of antibody transfer in birds. Similarly high estimates of repeatability have been shown for several key egg constituents that play an important role in avian development such as maternal yolk hormone levels, both across breeding attempts [25,26] and between years [27]. It is unclear whether females that transfer high levels of one maternal component such as antibodies may also transfer high levels of other key egg components in general. This would warrant further investigation to test if some females generally transfer more of all constituents or whether trade-offs occur (for example, more antibodies may be needed to balance hormonal immuno-mediated effects [28]).

The mechanism underlying differences in the ability of females to transfer specific antibodies remain unclear. Transfer of maternal immunity in birds is a two-stage process. Firstly low levels of antibodies are transferred to the yolk as it develops and at oogenesis, then there is a sudden influx of antibodies and other yolk components in the last few days prior to egg formation and laying [10]. This is believed to be receptor mediated and individuals may differ in the number and effectiveness of this transfer process. However, the transfer of antibodies also appear to be directly correlated to the increase in size of yolk as an egg is formed [10] so investment may be tied to allocation of other constituents laid down in the last few days of egg formation (though we foud no evidence that a female’s average egg size is related to her general level of transfer). There may also be variation in transfer levels based on variation in antibodies produced; for example, surprisingly, quail have been shown to transfer higher levels of chicken IgY than quail IgY in experimental manipulations [29]. How the molecules of IgY may vary between and within individuals remains unclear and is something seldom considered by immunologists as detection techniques tend to target specific known parts of the molecule making this hard to address (J. Allen pers.com). In birds, eggs are produced on a 24-hour cycle so as one egg is laid, the yolk for the next day’s egg has just been formed and packaged and is awaiting fertilization in the reproductive tract and the egg for the day after is still sequestering yolk constituents in the ovary. This sequential pattern of egg production over a series of days explains why eggs vary in their antibody level over the course of an immune response. Understanding these parts of egg development may be important for determining what ultimately controls variation in transfer of maternal antibodies and other yolk components.

In this study the ratio of antibodies transferred to eggs had a significant negative relationship with body condition. We examined whether the effect may arise from low condition females producing smaller eggs but transferring similar amounts of antibody with the result that yolk concentrations of antibodies could be higher. However, this did not appear to explain this result in this case; when yolk size was taken into account in our models, we found no interaction between maternal condition and yolk volume on the amount of antibody transferred. In normal infections a negative relationship between immune response and transfer and maternal condition might not be surprising as females experiencing high levels of infection may be both more likely to produce and transfer a large immune response while simultaneously suffering the costs of associated with infection [30]. However, by purposefully selecting inactive vaccines with no associated pathology we can rule out this possibility. Furthermore by establishing mothers from a clean colony of birds we can also rule out any effects of exposure history. The negative relationship in the level of antibodies transferred and condition might therefore appear counterintuitive as it is often assumed producing an antibody response is costly and that individuals in better condition can “afford” to invest more in transferring egg antibodies to their offspring [31-34]. However, a negative relationship between maternal condition and transfer has also been reported in other systems; in kittiwakes for example, non-food-supplemented females transferred more resources, including antibodies, to offspring compared to food supplemented females [32]. Other studies in birds, however, have found improving maternal condition can increase egg antibodies transfer [32], while others have found no effect of diet manipulation on immune transfer [35] so the relationship may vary depending on the conditions faced. Indeed changing environmental conditions have been shown to reverse the impact of infection for different offspring in some avian species [36]. Alternatively and perhaps more likely given our housing conditions, our findings may simply reflect differences between females that correlate with both their propensity to transfer antibodies and their mass to size ratio. For example, differences in maternal condition are likely to reflect differences in an individual’s early conditions and their ability to compete and gain food which could in turn affect either the development of their receptor based system or their ability to utilize this mechanism to maximize transfer. In contrast, female cumulative antibody level alone does not have a relationship with body condition scores, suggesting that female’s ability to produce an antibody response is not confined to the same factors as female’s propensity to transfer antibodies to eggs. We also found no trade-off between levels of allocation and levels of overall reproductive investment to suggest females might be trading off costs of antibody production or transfer with other costly aspects of reproduction in this system though birds were not under severely food limited conditions (10% lower feed than adlib).

We have demonstrated that the propensity to transfer protection to offspring can be consistent within individuals but vary between individuals. Could there also be adaptive active allocation over and above these effects? There is certainly evidence that levels of antibodies vary with laying sequence [37], brood value [33] and sex of offspring [13]. However, the single most important factor affecting how much antibody was found in the eggs was at what point in the immune response a female was at when a particular egg was laid. For example, if a clutch of eggs was collected from a female in the initial phase of her immune response – perhaps up to 10 days post-challenge – she would likely show a trend of laying eggs with higher levels of antibodies towards the end of the clutch as antibody levels would be increasing on a daily basis and oocytes for successive eggs develop in 24 hour cycles and are laid approximately 24 hours apart. However, if we had collected a clutch of eggs slightly later, we might find females lay eggs with fewer antibodies in the end of the clutch as these are being formed at the time point just after the peak of her immune response, so subsequent eggs would sequester declining levels of antibodies from the female’s circulating blood. Females show considerable variation in the magnitude and timing of peak responses, thus making it difficult to predict what an average response in response to challenge (or to natural levels of infection) might be without corresponding measure of female antibody levels over the same time period. In natural systems infection history may not be known and we have demonstrated that females can be very variable in both the magnitude and timing of their immune responses even following a controlled experimental challenge; collecting maternal antibody level throughout a clutch in these types of study would therefore be very informative. Furthermore, the discovery that the distribution of the sexes [38] and extra- pair paternity (EPP) offspring [39] are often non-randomly distributed within a clutch demonstrates the need to ensure these correlated antibody levels with the point in a female’s response are considered too. This does not necessarily mean that these patterns are not adaptive – directing particular offspring to a particular part of clutch that may receive more or less antibodies may in itself be an adaptive strategy and a mechanism by which allocation could be adjusted. However, distinguishing between these alternatives is crucial in terms of our understanding of the mechanisms underlying these effects and their implications for offspring fitness as they develop.

## Conclusions

We have shown there is substantial variation between females in the proportion of their own antibodies they transfer to their offspring and that this is unrelated to the overall magnitude of an individual’s own immune reponse. This has important implications as it suggests that both a female’s propensity to produce a particular level of immune response and her ability to transfer it to offspring may be two separate traits open to manipulation or selective processes. Estimating the transfer component of maternal immunity as a separate trait may therefore allow more informed decisions to be made to increase the production, health and welfare in domesticated animals exposed to disease and increases our understanding of why different offspring may vary in their responses to infection and the consequences maternal traits may have on pathogen host dynamics across generations.

## Materials and methods

### Study system: establishing the maternal generation

Chinese painted quail (*Coturnix chinensis*) are small game birds in the Phasianidae family and have been noted as being an ideal candidate for laboratory experiments due to their small size (40-85g), low aggression and high reproductive success in captivity [40]. *Coturnix* quail are also a commercial species and share many traits with other closely related poultry and are particularly valued for the egg production. The study was conducted using a colony of Chinese painted quail established at the University of Edinburgh to provide parental birds which have no previous exposure to common pathogens. In their natural environment (as wild or domesticated birds) individuals would most likely be in contact with and responding to a range of pathogens. Booster effects of repeated exposure to the same disease and secondary costs of dealing with infection aside from the mounting of an antibody response can therefore impact on measures of both antibody production and measures of host fitness. We therefore controlled for these effects by using inactive as opposed to live vaccines and a clean colony free of the pathogens of interest. By removing these potential confounding effects we were also able detect effects using a smaller sample size to address the specific questions of the study (in line with the 3Rs strategy) and to follow the effects of vaccination on the level of antibody response and transfer over time.

To establish the parental generation, eggs were collected from multiple commercial breeders around the UK and sprayed with Ambicide™ (1% dilution) prior to incubation to prevent any transfer of common environmental pathogens into the colony. Eggs were incubated under standardized conditions (37-38 °C and 40-50% humidity rising to 70% prior to hatching) then brooded for 24 hours prior to transfer to communal cages. Chicks were fed *ad libitum* and kept in large (2,400 × 500 × 375 mm) mixed sex cages (14 birds per cage) until they reached sexual maturity. Cages consisted of wood shavings, multiple feeding stations, covered areas and sand baths to allow birds to follow their full repertoire of natural behavior. A heat lamp was provided in one corner until 10 days post-hatching. Birds were ringed to allow individual identification at six weeks of age. Standard biosecurity measures to maintain a pathogen free flock were in place throughout the duration of colony establishment and the experiment.

### Experimental protocols

Two weeks prior to the start of the experiment, birds were moved into breeding groups in which they remained for the duration of the experiment. Birds were housed in cages (800 × 500 × 375 mm) in a ratio of three females to one male. Each cage was lined with wood shavings, and contained a nest area for each female and a communal sand bath. Adult birds were maintained on a photoperiod of 16 h:8 h L:D and on a diet of Haith’s finch seed, EMP, Prosecto Insectivorous and oystershell in a mix of: 20% protein, 2.5% calcium and 77.5% seed for the duration of the experiment. Total feed per cage was 71.6g based on 17.9g of feed per bird, which is 10% less than how much an adult laying female consumed *ad lib* over a 24hour period (unpublished data).

Once laying commenced, females were observed continuously during the egg-laying window to identify the color morph of each female to enable eggs to be assigned to individual females. Eggs are highly polymorphic in this species in both background color and in the presence/absence and type of markings. No females with similar egg markings were housed in the same cage. Eggs were removed on the same day they were laid to induce females to continue laying over the course of the experiment. Biometrics of females (total body mass (g), tarsus length (mm)) were measured and baseline blood samples were collected on day 0 prior to treatment, along with tarsus and mass measurements.

### Vaccine selection

The response of mothers and subsequent levels of transfer were monitored for two different types of vaccine effective against diseases that are important in both domesticated and wild bird populations. The first was a vaccine (Paramyxo P20, Intervet) against the viral disease NDV (Newcastle Disease Virus); this is a notifiable disease known to infect 200 bird species and has high virulence in poultry [41]. The second was a vaccine (SalenvacT, Intervet) against the bacterial pathogen *Salmonella*; a monitored pathogen of poultry breeding flocks and hatcheries by the European Union (EU) under the Zoonoses Directive 2003/99/EC [41]. Bacterial and viral challenges utilize different arms of the immune system when generating a response in the mother. Both vaccines had previously been demonstrated to produce antibodies that were effective at protecting offspring; NDV [42] and *Salmonella*[43].

### Challenge of treatment groups

Each female in each cage was randomly assigned to one of three treatment groups: a negative control group was injected with 0.1ml of phosphate-buffered saline (PBS) (n = 19), the bacterial challenge group was challenged with 0.1ml inactivated *Salmonella* vaccine (SalenvacT, Intervet) (n = 19) and the viral challenge group was challenged with 0.25ml inactive Newcastle Disease virus (NDV) (Paramyxo P201 Intervet) (n = 18). Each cage therefore contained one bird of each treatment, controlling for cage effects. Blood samples were taken immediately prior to challenge then every 4 days up until 39 days post-vaccination. Samples were collected via puncture of the metatarsal vein using a sterile needle and collection of the blood using 100μl capillary tube. Blood samples were centrifuged on day of collection at 13,400 rpm for 10 minutes and serum collected and stored at -20°C until antibody analyses were conducted. All work was conducted under Home Office License (No PPL 60-4115), with full ethical approval from the University’s ethical committee and with veterinary supervision throughout.

### Measurements of maternal traits

The mass and condition of all females were measured on all blood-sampling days. As this was an experimental set up with birds kept under standardized conditions, body condition was calculated by mass/tarsus length^3^ (see [44] for discussion on the merits of different body condition indices). Eggs were generally laid in the early afternoon and collected on a daily basis throughout the experiment.

### Repeatability of response over different treatments

A subset of individuals was re-vaccinated at 42 days post first treatment (n = 7). Four SalenvacT individuals were vaccinated additionally with 0.25ml NDV at day 42 and three NDV individuals were additionally vaccinated with 0.1 ml SalenvacT. Blood samples and condition scores continued to be collected at four-day intervals and eggs were collected daily from these birds until 76 days post-first vaccination.

### Egg measurements and antibody extraction

Egg length, width and mass were recorded at the time of collection, eggs were then frozen at -20°C for antibody analysis. Yolk mass was recorded after defrosting at the time of antibody extraction.

Antibodies were extracted from egg yolk following the protocol from Mohammed et al. (1986) [45]. The homogenized yolk was diluted 2:1 in Phosphate- buffered saline (PBS) and vortexed for 2 minutes. Chloroform was then added to the egg yolk/PBS mixture at a 1:1 ratio, and vortexed for a further 2 minutes. The mixture was then centrifuged on 13,400 rpm for 10 minutes. After this time the mixture separates into three layers; a top layer containing PBS and supernatant (used for antibody analysis), a deposit of fatty lipids in the middle layer and an organic phase containing chloroform and carotenoids in the bottom layer.

### Antibody analysis: enzyme-linked immunosorbent assay (ELISA)

Specific enzyme-linked immunosorbent assays (ELISAs) were performed for treatment and control groups to detect IgY. For the SalenvacT treated individuals a *Salmonella*-specific ELISA test was performed using FLOCKTYPE® *Salmonella* ELISA kit (Labor Diagnostik Leipzig, Germany). For the Paramyxo P201 treated individuals, a Newcastle disease virus-specific ELISA test was performed using FLOCKTYPE® recNDV ELISA kit (Labor Diagnostik Leipzig, Germany). Both kits were manufactured for chicken serum and plasma but quail antibodies are also detected and a number of studies have used anti chicken antibody to detect antibodies in quails [29,35,46]. Previous pilot work had established the appropriate dilutions to ensure antibodies lay within the bounds of the detectable range of the ELISA kits and dilution curves confirmed these lay within the linear part of the test.

Yolk extractions were diluted 1:62 and blood samples were diluted 1:124 with the buffer provided. The optical density (OD) of the resulting solution was read in a spectrophotometer at 450nm immediately after stopping the reaction, and corrected for using positive and negative controls supplied in the kit. To estimate the repeatability of the method, one sample for each of the treatment groups was tested on all of plates for each ELISA kit. The estimated repeatability within plates (98.35%, F_16,17_ = 0.898) and between plates (98.21%, F_16,17_ = 0.770) per kit was high. Antibody titre was calculated using the mean values (MV) of the measured optical density (OD) for the negative control (NC) and positive control (PC). The ratio sample to mean PC was then calculated using the following equation.

$$S/P\;\mathrm{ratio}=\frac{OD_{\mathrm{sample}}‒\mathrm{OD}{\left(\mathrm{MV}\right)}_{\mathrm{NC}}}{\mathrm{OD}{\left(\mathrm{MV}\right)}_{\mathrm{PC}}‒\mathrm{OD}{\left(\mathrm{MV}\right)}_{\mathrm{NC}}}$$

Control individuals had readings of 0.0491 ± 0.0447_2SD_ (*Salmonella* ELISA plate) and 0.029 ± 0.067_2SD_ (NDV ELISA plate). Any serum S/P ratio of less than 0.1 was treated as a negative antibody response (within the confidence intervals of the control groups antibody titre values) resulting in 9 birds (1 SalenvacT, 8 NDV) being excluded from the study. Seven birds (4 SalenvacT, 3 NDV) did not produce a full sequence of eggs or ate their eggs before they could be collected and were also excluded from the study. We were therefore able to collect a full sequence of blood and egg antibody levels of the course of a laying sequence for 12 control females, 14 bacterial challenge females and 7 viral challenge females.

### Statistical analyses

Statistical analyses were performed in R (version 2.10.1). We fitted mixed models using the lme4 package. We investigated the effects of a range of factors on the amount of antibody transferred to eggs using linear mixed effects models and linear models and calculated repeatability estimates from the intraclass correlation coefficient. Female antibody titre and egg antibody titre were log transformed to achieve normality. Treating egg antibody level as a dependent variable, we investigated the effects of treatment (vaccination against NDV vs Salmonella), females’ circulating antibody level, yolk mass, female condition and number of days since challenge on the amount of antibody transferred to the embryonic environment. Female ID was included in all repeated measures models as a random effect. Blood samples were collected from females every 4 days. To examine how egg antibody levels varied with a female’s antibodies over the course of the antibody response we ran a smoothing spline on the female’s antibody response to get predicted values for days where blood samples were not collected. Lambda was set at 0.1 to maximize a close fit to the actual data available.

To explore the relative transfer of antibodies within a challenge and across challenges we controlled for the effect of variation between mothers in their own antibody responses to challenge by calculating the ratio of circulating antibody transferred to the egg (egg antibody/female antibody) and using this as our dependent variable. This is a measure of the relative amount of antibodies a female transfer regardless of her own response. This allowed us to investigate how this ratio of antibodies transferred to the second challenge in relation to the first challenge, in this model we also explored which was treatment was given first to determine if there was any order effect.

## Competing interests

The authors declare that they have no competing interest.

## Authors’ contributions

CMC carried out the experimental work, immunoassay and antibody extraction work. KAH participated with experimental work and egg separation and morphometric data collection. VS participated in the exploration of data and discussion on manuscript. CMC and EJAC were both involved with design of the study and performed the statistical analysis. EJAC conceived of the study, and participated in its design and coordination and helped to draft the manuscript. All authors read and approved the final manuscript.
